# Diagnosis of animal trypanosomoses: proper use of current tools and future prospects

**DOI:** 10.1186/s13071-022-05352-1

**Published:** 2022-06-27

**Authors:** Marc Desquesnes, Alireza Sazmand, Marisa Gonzatti, Alain Boulangé, Géraldine Bossard, Sophie Thévenon, Geoffrey Gimonneau, Philippe Truc, Stéphane Herder, Sophie Ravel, Denis Sereno, Etienne Waleckx, Vincent Jamonneau, Philippe Jacquiet, Sathaporn Jittapalapong, David Berthier, Philippe Solano, Laurent Hébert

**Affiliations:** 1 UMR INTERTRYP, French Agricultural Research Centre for International Development (CIRAD), 31076 Toulouse, France; 2 INTERTRYP, IRD, CIRAD, University of Montpellier, Montpellier, France; 3 National Veterinary School of Toulouse (ENVT), 23 chemin des Capelles, 31000 Toulouse, France; 4 Department of Pathobiology, Faculty of Veterinary Science, Bu-Ali Sina University, Hamedan, 6517658978 Iran; 5 Department of Cell Biology, Simón Bolívar University, Caracas, 1080 Venezuela; 6 UMR INTERTRYP, CIRAD, Bouaké, Côte d’Ivoire; 7 Pierre Richet Institute, National Public Health Institute, BP 1500 Bouaké, Côte d’Ivoire; 8 UMR INTERTRYP, CIRAD, 34398 Montpellier, France; 9 UMR INTERTRYP, CIRAD , Dakar, Senegal; 10 National Laboratory for Livestock and Veterinary Research, Senegalese Institute on Agricultural Research (ISRA), BP 2057, Dakar, Hann Senegal; 11 IRD, UMR INTERTRYP, University of Montpellier, Montpellier, France; 12 Regional Research Centre Dr. Hideyo Noguchi, Autonomous University of Yucatán, Mérida, Yucatán Mexico; 13 Faculty of Veterinary Technology, Kasetsart University, Bangkok, 10900 Thailand; 14 Physiopathology & Epidemiology of Equine Diseases Unit (PhEED), Laboratory of Animal Health, Normandy Site, French Agency for Food, Environmental and Occupational Health & Safety (ANSES), Rd 675 Les Places, 14430 Goustranville, France

**Keywords:** Integrative trypanosomosis diagnosis, Pan-trypanosome ELISA, POCD, PSR, Trypanosomosis, Trypanosome

## Abstract

Reliable diagnostic tools are needed to choose the appropriate treatment and proper control measures for animal trypanosomoses, some of which are pathogenic. *Trypanosoma cruzi*, for example, is responsible for Chagas disease in Latin America. Similarly, pathogenic animal trypanosomoses of African origin (ATAO), including a variety of *Trypanosoma* species and subspecies, are currently found in Africa, Latin America and Asia. ATAO limit global livestock productivity and impact food security and the welfare of domestic animals. This review focusses on implementing previously reviewed diagnostic methods, in a complex epizootiological scenario, by critically assessing diagnostic results at the individual or herd level. In most cases, a single diagnostic method applied at a given time does not unequivocally identify the various parasitological and disease statuses of a host. These include “non-infected”, “asymptomatic carrier”, “sick infected”, “cured/not cured” and/or “multi-infected”. The diversity of hosts affected by these animal trypanosomoses and their vectors (or other routes of transmission) is such that integrative, diachronic approaches are needed that combine: (i) parasite detection, (ii) DNA, RNA or antigen detection and (iii) antibody detection, along with epizootiological information. The specificity of antibody detection tests is restricted to the genus or subgenus due to cross-reactivity with other *Trypanosoma* spp. and Trypanosomatidae, but sensitivity is high. The DNA-based methods implemented over the last three decades have yielded higher specificity and sensitivity for active infection detection in hosts and vectors. However, no single diagnostic method can detect all active infections and/or trypanosome species or subspecies. The proposed integrative approach will improve the prevention, surveillance and monitoring of animal trypanosomoses with the available diagnostic tools. However, further developments are required to address specific gaps in diagnostic methods and the sustainable control or elimination of these diseases.

**Graphical Abstract:**

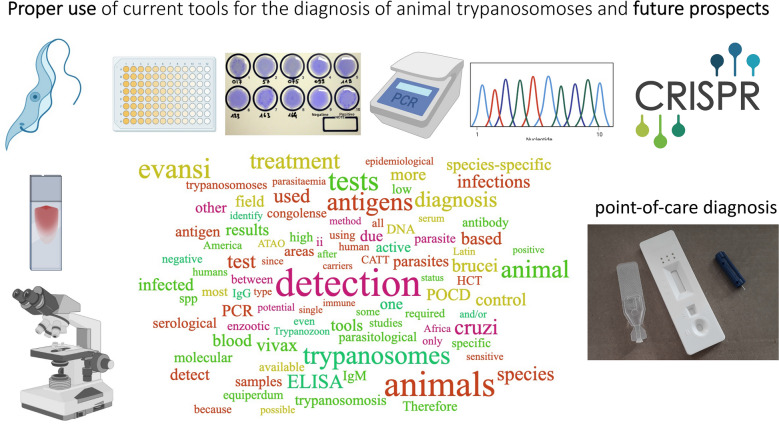

## Background

The *Trypanosoma* spp. and trypanosomoses included in this review are mostly related to salivarian trypanosomes, including: (i) the complex of *Trypanosoma congolense*, *T. brucei brucei*, *T. vivax* and *T. simiae*, which cause the disease widely known as nagana (i.e. animal African trypanosomosis [AAT]); (ii) *Trypanosoma brucei gambiense* and *T. b. rhodesiense*, which cause the disease widely known as sleeping sickness (i.e. human African trypanosomiasis [HAT]); (iii) *Trypanosoma evansi*, which causes surra; and (iv) *Trypanosoma equiperdum*, which causes dourine. However, it also includes a stercorarian trypanosome: (v) *T Progressive Control Pathway (PCP) cruzi*, the aetiological agent of Chagas disease (also referred to as American trypanosomosis).

Trypanosomoses are a serious animal health concern, causing significant disease in livestock and domestic animals in Africa, Asia and the Americas. In addition, the international trading of live animals makes them a permanent threat to Europe and Australia [1, 2]. Therefore, a reliable and comprehensive diagnosis is the basis for epidemiological investigations, individual diagnosis, prevention, treatment, monitoring, surveillance, control and international trading.

Our previous review focussed on the intrinsic characteristics, advantages and drawbacks of the methodologies available for animal trypanosomosis diagnosis, irrespective of the users’ needs [3]. In the present review, we focus on the proper use of currently available tools, based on the information they provide, to gain the maximum benefit in the various epidemiological circumstances in which they are required. When presenting their limitations, we also touch on further possible developments.

First of all, we need to define the terms used, as words with double meanings are a source of confusion and misunderstanding. In mammals (or other animals, such as birds), the term “trypanosomosis” (plural “trypanosomoses”), derived from the name of the causative agent “trypanosom-”, and the suffix “-osis” (plural “-oses”)”, meaning “disease(s)”, has more than one meaning, covering a large panel of parasitological, clinical and epidemiological situations. Two situations can be considered: (i) “trypanosome infection”, which refers to a past or ongoing infection due to one or more causative agents belonging to the genus *Trypanosoma*; and (ii) “trypanosome-associated sickness” (infection + disease), whereby an animal is actively infected by one or more trypanosome(s), and has clinical signs. Current diagnostic tools are unable to distinguish between these two situations. For example, in a highly enzootic area, and in the absence of a general control project, the systematic treatment of all “animals carrying anti-trypanosome antibodies” (up to 80% of the animals in some areas) would be a waste of money. Moreover, it may select chemo-resistant strains [4]. A cost-effective treatment-decision test would instead require identifying only “sick animals” [5]. When parasite elimination is the objective, as in the case of the advanced stages of a Progressive Control Pathway (PCP)[6], active infection by itself is the rationale for treatment. In addition, non-pathogenic trypanosomes such as the ubiquitous *Trypanosoma theileri* must be identified.

Therefore, in this review we will first define individual host status with the available diagnostic tools, such as “non-infected”, “actively infected”, “infected and sick”, “asymptomatic carrier”, “species-specifically infected”, etc. Additionally, we will look at parasite detection in vectors. This review presents a broad and global perspective encompassing diagnostic tools, with short-, medium- or long-range objectives, in the framework of a variety of scenarios or situations: (i) enzootic areas, (ii) sporadic cases, (iii) countries or regions declared parasite free and (iv) potential zoonotic occurrences. *In fine*, realistic prospects and strategies for developing new tools will be discussed.

## Individual status inferred from current diagnostic tools

There are critical periods in the course of an infection that are directly associated with the reliability of a diagnosis. For instance, at the onset of an infection, during the first 2–3 weeks after a *Trypanosoma* infection, any samples analysed by parasite or antibody detection methods may provide false-negative results. Likewise, parasite detection tests in asymptomatic carriers may not reveal the infection, while antibody detection tests would in this case be effective. Conversely, after curative treatment, it may not be possible to ascertain the presence or absence of infection due to the persistence of antibodies in the serum for months [7]. Serial samplings may address such situations.

**Fig. 1:**
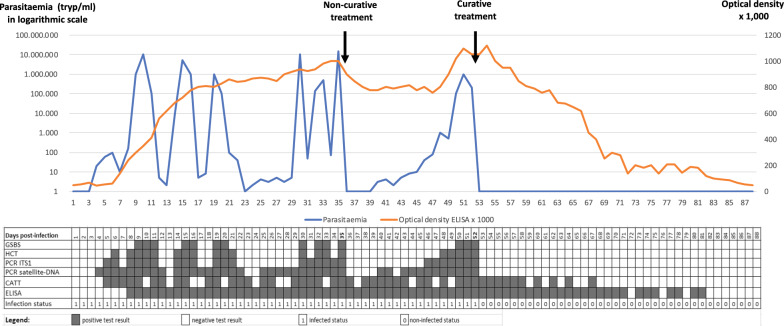
Parasitaemia in trypanosomes per millilitre (tryp/ml; blue curve) and optical density × 1000 in ELISA (orange curve), modelled in an animal infected by a trypanosome (*Trypanosoma evansi* in this case) on D0 (Day 0), receiving 1 non-curative treatment on D35, and one curative treatment on D52. See Abbreviation List for the full description of each abbreviation

One or more diagnostic tools need to be used during single or serial sampling to enable a conclusion to be drawn or inference made on a mammal’s status regarding trypanosome infection, whether “non-infected” or “actively infected”, which can either be an “asymptomatic carrier” or a “sick carrier”. As was previously discussed [3], the test specificity can vary, the primers must be selected according to subgenus, species, type and subspecies, and the test results will still remain inconclusive for single or mixed infection status. By using one or more diagnostic tools for serial examinations, it will be possible to differentiate current (parasite ± antibodies) from past infections (antibodies only). Possible outcomes of diagnostic tests regarding the infection and immune statuses are given in Fig. 1.

This figure represents test outcomes from infected animals (*T. evansi* in cattle, for example) receiving non-curative and curative treatments. Based on the results gathered during experimental infections, we modelled the follow-up of an animal infected on day 0 (D0). In this model, the positive thresholds for the diagnostic techniques were set up as follows: Giemsa-stained thin blood smear (GSBS): 10,000 trypanosomes per millilitre (tryp/ml); haematocrit centrifugation technique (HCT): 100 tryp/ml; PCR-internal transcribed spacer 1 (ITS1): 30 tryp/ml; satellite-DNA PCR: 3 tryp/mll, and enzyme-linked immunosorbent assay (ELISA) optical density (OD) = 0.2. A non-curative treatment was given on D35; this was followed by an “aparasitaemic” period, but the OD in ELISAs remained high. A relapse of the infection was detected from D40 onwards by satellite-DNA PCR, followed by PCR-ITS1 (D46), HCT (D48) and GSBS (D50). A curative treatment on D52 was followed by an “aparasitaemic” period then a progressive decrease, based on OD, until it became negative from D82 onwards. A negative seroconversion 30 days after curative treatment is short; longer periods are generally observed in the field, particularly in older animals having undergone multiple infections. The inconsistency in detection of early infections by the card agglutination test for trypanosomosis (CATT) can be attributed to the presence of immune complexes [8]. On the other hand, it turns negative before the ELISA due to the short half-life of immunoglobulin M (IgM).

### Establishment of a “non-infected” status

Due to fluctuating parasitaemia, it is not always possible to demonstrate the presence of trypanosomes in infected animals. Therefore, negative results from parasitological and/or molecular techniques are insufficient to establish a “non-infected” status. Repeated non-detection of antibodies after 1 month (> estimated incubation period of 2–3 weeks) is a stronger criterion.

Immunoglobulin G (IgG) produced against trypanosomes can be detected in the serum from 2 to 3 weeks after infection. According to the recommendations of the World Organisation for Animal Health (WOAH, formerly known as OIE), this is especially true with ELISA plates coated with a complete native antigen, such as whole-cell lysate-soluble antigens (WCLSA) [9, 10]. IgG detection is thus a reliable method for establishing a “non-infected” status in most cases. However, negative parasitological, molecular and serological results are needed twice at a 1-month interval [10].

Nonetheless, a serological test may give a false-negative result in the case of *Trypanozoon* infections that display occasional extravascular foci, whereby the parasite is not in contact with the immune system [11]. This was hypothesised in the *T. evansi* camel outbreak that occurred in France [1]. However, such a situation is probably rare and limited to *T. evansi* in camels. In most cases, accurate indications are obtained from an ELISA, as demonstrated in several validations among different host species. All of these studies agreed on the high sensitivity and reliability of IgG detection by ELISA using WCLSA of trypanosomes [12–19].

In conclusion, with the exception of surra in camels, an unequivocal “non-infected” status can be established (in cattle, buffaloes, horses, sheep, goats, dogs, etc.) if negative results are obtained in a quarantine context, twice at a 1-month interval [9, 10], using: (i) HCT; (ii) one or more molecular detection tests (PCR) selecting the most suitable primers; and (iii) one or more WCLSA ELISA(s), selecting the most suitable species (ELISA *T. vivax, T. congolense, T. brucei/T. evansi* and/or *T. cruzi* in Latin America), according to the geographical area and the epizootiological situation [3]. Furthermore, due to its cross-reactivity with all pathogenic mammalian trypanosomes (*T. brucei*, *T. equiperdum, T. vivax, T. congolense* and *T. cruzi*), the *T. evansi* ELISA test would be an ideal candidate for such screening [17, 20–22].

In animals for which no species-specific anti-IgG conjugate is commercially available, protein A-conjugate may be used. However, results using this conjugate have not been fully validated and standardised due to a lack of reference sera from non-infected and infected animals. Consequently, the status of such animals regarding trypanosomosis cannot be certified.

### Detection of an “active infection”

Microscopic examinations of GSBSs and buffy coat [23] are quick and cheap ways of detecting active infections. These methods remain the most usual choice in enzootic areas. However, they lack sensitivity and require a minimal level of equipment and skill, which are not always available in the field. Nevertheless, a positive result indicates a parasitaemia > 50–100 tryp/ml, which reflects the immune system’s inability to control the infection and should lead to a treatment decision.

Other parasitological methods, such as the kit for in vitro isolation (KIVI) [24] or the mouse inoculation technique (MIT) (although raising ethical issues), remain the most efficient techniques for parasite isolation [8]. They can be used to demonstrate the parasite’s presence, but also allow further characterisation and storage of field isolates. When applied to diagnosis, they are more sensitive than HCT and GSBS [25], but are relatively expensive and time-consuming. Still, they are helpful for trypanosome isolation during an outbreak in a previously non-endemic area [26] or for high-value animals (racehorses, zoo animals, etc.). The added value of the MIT is clear for *Trypanosoma* species such as *T. evansi* and *T. brucei* that multiply readily in rodents, but for other *Trypanosoma* species, such as *T. congolense* and *T. vivax,* results are inconsistent and, in general, negative for *T. equiperdum* [27].

Molecular detection of trypanosomes through PCR was a real breakthrough in the development of trypanosome diagnostic techniques in the 1990s [28–30]. PCR improved the sensitivity for detecting active infections and significantly improved specificity, at various taxonomic levels. However, molecular methods have critical limitations: (i) they leave cases with low parasitaemia or non-circulating parasites undetected [31, 32]; (ii) they are limited to fully equipped laboratories with skilled technicians; (iii) positive results are conclusive of active infection (leaving aside the fact that DNA may still be detected 24–48 h after curative treatment [33]), but negative results are not; and (iv) there is a significant delay between the time of sampling in the laboratory and the delivery of results, so animals can be out of reach by the time the veterinarian, vet technician or owner receives the results. Loop-mediated isothermal amplification methods (LAMP) applied to parasite DNA can mitigate such drawbacks. Although they were claimed to be efficient and applicable in the field [34–36], this has never really been the case. The new polymerase spiral reaction (PSR) method [37] may be suitable for field diagnosis in real time, but it still requires comprehensive field validation. Finally, the new and promising spliced-leader RNA (SL-RNA) detection method is applied to a short and conserved RNA sequence linked to the 5’ end of each trypanosome pre-messenger RNA (mRNA) [38]. Still, its implementation requires expensive equipment for quantitative PCR (qPCR) and skilled personnel [39].

Although antibody detection indicates contact between the host and parasites, it does not confirm active infection, especially as IgG persists several weeks after treatment or self-cure (2–4 months). IgM is produced early and has a short half-life (1–3 months) [8, 40], and is associated with recent infection or recent parasite circulation. IgM detection has a good positive predictive value for detecting active infections, while IgG tests detect an “established infection”. However, IgM immune complexes are captured by phagocytic cells in the serum of actively infected animals and, consequently, IgM detection can give false-negative results [8]. Overall, implementing IgM and IgG detection in a herd showing signs of active infection (positive HCT or PCR) can help identify infected animals, but these methods not suited to detect active infection on their own.

In summary, at the individual level, an active infection can only be established with parasitological (HCT, GSBS, MIT, etc.) and/or molecular tools (PCR, LAMP, PSR, RNA detection, etc.). However, once the infection is confirmed in one or more animals in a group, seropositivity may be considered sufficient by a primary care veterinarian to decide on eliminating parasites, even in apparently healthy animals. Conversely, a “sickness treatment-decision strategy” requires evidence that clinical signs are linked to active infection (see following sections).

### Sick or healthy status and treatment decisions

As discussed earlier [3], in enzootic areas (and in the absence of an elimination programme), before deciding on a treatment, a distinction should be made between the “infected and healthy animal” (asymptomatic carrier) and “infected and sick animal” statuses. A meta-analysis aggregation including averaging the results of 180 studies on cattle trypanosomosis in 19 enzootic African countries [41] showed a low prevalence of 15.1% (95% confidence interval: 13.2–17.1). Nevertheless, on a smaller scale, in enzootic areas of Burkina Faso, Cameroon and Ghana, > 50–70% of the cattle are seropositive [42–44]. In such areas, a high percentage of cattle are asymptomatic carriers. Therefore, unless there is an ongoing disease elimination programme, despite being seropositive, these animals do not need treatment. A high seropositivity level makes it difficult (if different from tossing a coin) to draw a causal relationship between the presence of anti-trypanosome antibodies and sickness due to trypanosome(s), even in animals with clinical signs compatible with trypanosomosis and other diseases [45]. There are no markers of “trypanosome sickness”. Although a low haematocrit value can help, the sickness may be caused by other agents, such as ticks, *Haemonchus* spp., haemoparasites, etc. [46]. HCT is an excellent indicator because its low sensitivity indicates a medium to high parasitaemia, suggestive of “insufficient immune control”. Additionally, it may estimate anaemia (low packed-cell volume), which is undoubtedly sufficient reason for trypanocidal treatment.

As microscopes and centrifuges are rarely available in the field, a rapid antigen detection test would help evidence recent circulation of parasites, even with limited sensitivity. Like the *T. evansi* CATT, card agglutination tests for other *Trypanosoma* spp., based on IgM, would be of predictive value for trypanosome sickness and support treatment decisions.

### Can species and/or subspecies-specific diagnosis be established?

The ELISA offers a large panel of antigens with sensitivity close to or higher than 95% [15, 47, 48] and a high specificity in relation to other genera, such as *Anaplasma*, *Babesia,* etc. However, species specificity remains low and strong cross-reactions occur between the main animal trypanosomoses of African origin (ATAO): *T. vivax, T. congolense, T. brucei brucei*, *T. evansi* and *T. equiperdum* [17, 49]. These cross-reactions are due to common antigens shared by salivarian *Trypanosoma*, but even occur between taxonomically distant species such as *T. evansi* and *T. cruzi* [20], or *Leishmania* [20, 50, 51]. The species specificity of serological tools and therfore “seropositivity” is thus questionable. Fortunately, Megatrypanum such as *T. theileri* has been shown not to cross-react in an ELISA for trypanosomes [52, 53]. Consequently, ELISAs carried out with WCLSAs of salivarian trypanosomes are specific to “pathogenic *Trypanosoma* spp.”, but fail to identify them at the species level.

For ATAO control, in most cases−and especially in tsetse-infested areas−a species-specific diagnosis may not be necessary because the control tools (e.g. fly traps and trypanocides) are mostly identical, regardless of the salivarian *Trypanosoma* species involved. However, knowledge of the infecting *Trypanosoma* spp. is useful for adjusting the dose and trypanocide to be used. For example: (i) if *T. evansi* is identified, melarsomine hydrochloride is preferable; (ii) in a nagana area, identifying the species would allow the dose of diminazene aceturate to be adapted to 7 mg/kg for a *Trypanozoon* infection versus 3.5 mg/kg for an infection by *T. vivax* or *T. congolense*.

At the genus level, the co-infection status of an animal seropositive for *Trypanosoma* spp. or *Leishmania* spp. antibodies cannot be established using immunodiagnosis due to cross-reactions. In Latin America, cross-reactions should be suspected in studies involving *Trypanosoma* spp. (*T. evansi, T. vivax, T. cruzi, T. equiperdum*) and *Leishmania* spp. Similarly, caution is required with *Leishmania* and *Trypanosoma* serological studies in Asia and Africa. Indeed, in Latin America for example, *T. vivax* and *T. evansi* ELISAs may react or cross-react due to infection(s) by *T. viva*x, *T. evansi* and/or *T. cruzi*. This is especially so for horses and pigs when using a *T. evansi* ELISA [54, 55], but also for cattle [56] and buffaloes for both tests [57]. Additionally, *Leishmania* infections, which may be prevalent in reservoirs such as dogs, are sources of interference [58]. In such cases, the use of species-specific molecular tests and point-of-care diagnostics (POCD) such as recombinant polymerase amplification with lateral flow dipstick (RPA-LFD), recently developed in Mexico for *T. cruzi*, would be of great value [59]. Furthermore, the recent discovery of *Trypanosoma caninum* complicates the diagnosis of Trypanosomatidae infections in dogs [60, 61]. In the USA, where *T. cruzi* [62] and *Leishmania* [63] are prevalent in horses, interference in serological diagnosis should be suspected, and this situation may also occur in dogs [64].

In Africa, unlike the agents causing nagana, which are considered to be a unique complex entity (at least for their control), species-specific diagnosis may be needed when human pathogens are circulating in animals. It is essential to investigate animal reservoirs to control and eliminate human pathogens [65]. Molecular techniques are well suited for this purpose since they are sensitive and specific. However, PCR may fail to detect infection when primers target a single-copy gene or when using a sample with low parasitaemia. Nested-PCR or RNA detection methods can increase the sensitivity of these tests. Identifying *T. b. gambiense* and *T. b. rhodesiense* using subspecies-specific antigen detection would be another option. However, although precise tests may be developed, they would probably have low sensitivity (due to the inverse relationship between sensitivity and specificity), leading to uncertainty when diagnosing negative test results.

Effective and user-friendly tests able to distinguish between all parasites of the subgenus *Trypanozoon* at the species or subspecies level [66] either have not yet been developed or lack sensitivity. For example, tests to distinguish *T. evansi* (type A, type B, etc.) from *T. equiperdum* (type OVI, BoTat, etc.) are inconclusive because of polyphyly [66–69] or the low sensitivity of the method, which uses single-gene DNA detection tests. In addition, variations in mitochondrial DNA (mtDNA) content (kinetoplastic DNA composed of maxi- and minicircles) can be used to differentiate some *Trypanozoon* species [70, 71], but the fact that many *T. evansi* strains are deficient in kinetoplastic DNA (akinetoplastic) [72] limits the widespread use of these tools. Whichever serological test is used (CATT for *T. evansi* or any of the *Trypanozoon* ELISAs), in areas of mixed infections, when a positive result suggests a subgenus *Trypanozoon* infection, it should only be considered a “pathogenic trypanosome infection”. *Trypanozoon* identification at the subspecies level is possible with DNA-based methods, but at the expense of sensitivity. As an example, positive results are obtained with satellite DNA detection (TBR/NRP primers), which is highly sensitive for *Trypanozoon* parasites [28, 30] thanks to the 10,000–20,000 sequence repeats. However, specific primers targeting single genes like* SRA* (*T. b. rhodesiense*/*T. b. brucei*) or* TGSGP* (*T. b. gambiense*/*T. b. brucei*) [73, 74]) may be ineffective because of insufficient DNA in the sample. Therefore, when TBR primers provide positive results, the final result will remain inconclusive if single-gene PCRs are negative. PCR sensitivity for diagnosis can thus be ranked as follows: satellite DNA > moderately repeated genes (e.g. ribosomal DNA, ITS1) > single-gene DNA.

When using PCR, a positive test is considered conclusive and a negative one is not, so when the taxon *Trypanozoon* is detected in a sample, even if one subtaxon is confirmed (e.g. *T. evansi* using Rode *Trypanozoon* antigen-type [RoTat]1.2 primers), it will not be possible to detect another *Trypanozoon* (namely in this example *T. equiperdum, T. b. brucei, T. b. rhodesiense* or *T. b. gambiense*) also present in the sample. Thus, a reliable and specific species/subspecies diagnosis may never be established in mixed enzootic hosts and areas.

In conclusion, we urgently need reliable species-specific methods to detect trypanosome infections because of: (i) cross-reactions in antibody detection methods; and (ii) the poor sensitivity of current highly specific DNA-based methods. Even though the epizootiological context would suggest the most probable conclusion (e.g. a trypanosome infection in a horse in the USA is likely to be caused by *T. cruzi*, while in Asia it would most probably be due to *T. evansi*), reasonable assumptions may be made only in enzootic areas. Even so, these conclusions cannot be drawn for travelling animals, which could have been exposed to “out of context” parasites. Let us give some rather extreme but also realistic examples. A dog or a racehorse from Africa, which has spent some days in Mexico and then has tested seropositive to an ELISA for *T. brucei* upon arrival in France should be considered as a potential carrier of one or more of the following pathogens: *T. vivax, T. congolense, T. brucei* spp*., T. evansi, T. equiperdum* (for the horse only)*, T. cruzi* and *Leishmania* spp. [17, 20]. If the same animal has positive PCR results for *Trypanozoon*, this result confirms it as a carrier of *T. brucei* spp*.* and/or *T. evansi* and/or *T. equiperdum.* At the same time, it remains suspect for all the other taxa mentioned above due to the PCR’s limited sensitivity.

Although monospecific infections remain the most frequent (thus explaining why we qualified our examples as “extreme”!), to be precise and exhaustive, we must keep in mind the possibility of combined infections. Thus, the only short conclusion on the lack of specificity and sensitivity in trypanosome diagnosis is that once a pathogenic *Trypanosoma* (or *Leishmania*) infection is detected, it can hide co-infection(s) by any other pathogenic *Trypanosoma* (or *Leishmania*). Such an animal is then a confirmed case of a *Trypanosoma* infection, in addition to a suspected case of other infection(s) by members of the Trypanosomatidae family.

### Diagnosis of trypanosomes in insect vectors

Detecting trypanosomes in insect vectors is feasible for epidemiological studies and risk assessment, but the meanings and limits of any conclusion need to be further discussed.

Trypanosomes can be detected in the mouthparts, salivary glands, midguts, rectum or crushed whole insects, with different significance in terms of development and transmission (mature or immature stage of the cyclical development).

The microscopic observation of trypanosomes in insects does not allow identification of the species since the morphology of insect stages of trypanosomes is not characteristic; therefore, pathogenic trypanosomes may be confused with non-pathogenic ones such as *T. theileri* (cyclically transmitted by tabanids), or with insect commensals of the Trypanosomatidae family, such as Crithidia [75].

#### In mechanical vectors

Detecting trypanosomes in the mouthparts of a mechanical vector suggests only a potential for transmission since they can only survive there briefly (30 min to 2 h) [76, 77]. Consequently, detecting trypanosomes in the mouthparts or the midgut of a mechanical vector indicates that the insect fed on an infected animal, but not that the parasite was transmissible to a host. Such information is not particularly relevant; it is easier and more informative to detect trypanosomes in the blood of individual livestock than in blood that is “randomly collected” by an insect. Detecting trypanosomes in haematophagous insects acting as mechanical vectors is therefore of little practical value on livestock farms [75].

However, the situation is different in conservation areas. Since wild animals are difficult to trap or are subject to regulations forbidding their capture, the blood collected by haematophagous insects can be of great interest. Indeed, the detection of trypanosomes in mechanical vectors feeding on wild fauna will provide information on the presence and circulation of these parasites among wild animals and their potential role as reservoirs. In such cases, identifying the insect’s blood meal could provide other complementary information using ELISA [78] or molecular methods, such as PCR amplification of the cytochrome* b* mtDNA [79]. In addition, it is possible to obtain more information on the vertebrate host through molecular markers or using more recent multiplexed next-generation sequencing (NGS) methods, such as metabarcoding [80, 81]. The application of such methods would notably help to determine the potential circulation of the parasites between host species. However, detection of this kind is rarely implemented due to the low rate of fed insects entering insect traps. This rate could be increased through the use of ultraviolet (UV) light [82], although cost remains a limiting factor.

#### In cyclical vectors

The case of cyclical vectors of trypanosomes is quite different. When these insects (tsetse flies or triatomine bugs) are found to be “infected” by trypanosomes, the probability that they transmit the parasite is high because they remain permanently infective; their role in the disease epidemiology can thus be addressed and measured [83].

Parasitological methods were the primary tool in the past. They are based on the location of parasites in the vector (gut, salivary glands, proboscis, salivary secretion, faeces) rather than on parasite morphology, which is inconsistent at the insect stages [84]. Molecular techniques have since proved to be more reliable and valuable than parasitological methods for epidemiological studies [85], but contamination during insect dissection could affect detection. Molecular methods are also expensive, especially when considering the total number of PCR tests required per insect, as the number of insect organs is multiplied by the number of *Trypanosoma* species investigated [85]: 3 × 3 = 9 for (gut + salivary glands + proboscis) × (*T. vivax* + *T. congolense* + *T. brucei*), for example. One option is to dissect the insect and observe body parts through a microscope first, then proceed with PCR tests only for positive samples. Such methods were used to study *T. cruzi* circulation in Latin America in the framework of large-scale studies to identify biological, ecological and environmental variables associated with Chagas disease [86]. In Africa, they were used to identify pathogenic trypanosomes in tsetse flies [87].

Xenodiagnosis, previously used only for humans [88], is an exciting avenue to explore; it is a sensitive and specific tool to identify trypanosomes in animal and human hosts. Unfortunately, although used for *Leishmania* [89] and *T. cruzi* detection in Latin America [90], it has been limited to experimental infections for African trypanosomes [91].

In conclusion, the benefit gained from trypanosome detection in a mechanical vector is limited to wild animals or the interface between livestock and wildlife. More general information can be obtained from tsetse flies and triatomine bugs. Better information should be obtained when combining molecular identification and blood meal analyses [83, 92]. However, the cost of such studies is high since they require insect capture, identification and dissection, (multi-organ) × (multi-species) PCR diagnosis and multi-host blood meal identification [93].

## Diagnostic tools available and needed, in line with various epidemiological contexts

Diagnosis and treatment decisions for animal trypanosomoses are complicated by the fluctuating and frequently low parasitaemia in infected animals, as well as by the occasional absence of clinical signs. Moreover, numerous cross-reactions amongst *Trypanosoma* and *Leishmania* are a source of confusion in serodiagnostic test results. The information required and the means to collect it depend on the epidemiological situation, the scale of the study (population, individual), the objective of the test (treatment decision, infection status) and the availability of diagnostic methods (POCD or laboratory tests). An additional difficulty can appear in a “One Health” context: it may be necessary to treat animals infected by zoonotic trypanosomes, not because they are sick, but because they may be a threat to humans as a reservoir. This latter question is increasingly relevant in the context of eliminating transmission of *T. b. gambiense* (HAT) [94]. Finally, the treatment’s fully curative effect should be demonstrated in such a context.

The Pan African Tsetse and Trypanosomiasis Eradication Campaign (PATTEC) identified the need for more standardised evaluation methods [95]. The Food and Agricultural Organisation of the United Nations (FAO) further developed this by setting a PCP for ATAO, focusing on the African situation [6] and, very recently, on surra [96], although such a PCP has the potential for application in Latin America and Asia and for extension to other *Trypanosoma* spp., such as *T. evansi* [96].

Diagnosis plays a crucial role in reaching these ambitious goals. As for ATAO in Africa, the 21st Conference of the Regional Commission of the WOAH for Africa [97] proposed the self-declaration of trypanosomosis status, i.e. “free of trypanosomosis and tsetse-transmitted trypanosomes” for an area, or a whole country, which is indeed the most probable evolution in the future. Therefore, tools are needed to evaluate prevalence and detect carriers. More sensitive tools are also needed for application in occasional outbreaks in previously non-infected countries, animal trading or temporary movements. For each of these situations, we will first inventorise the needs, review the advantages and drawbacks of current diagnostic tools to highlight the best ways to use them and finally discuss prospects for developing new tools.

**Fig. 2:**
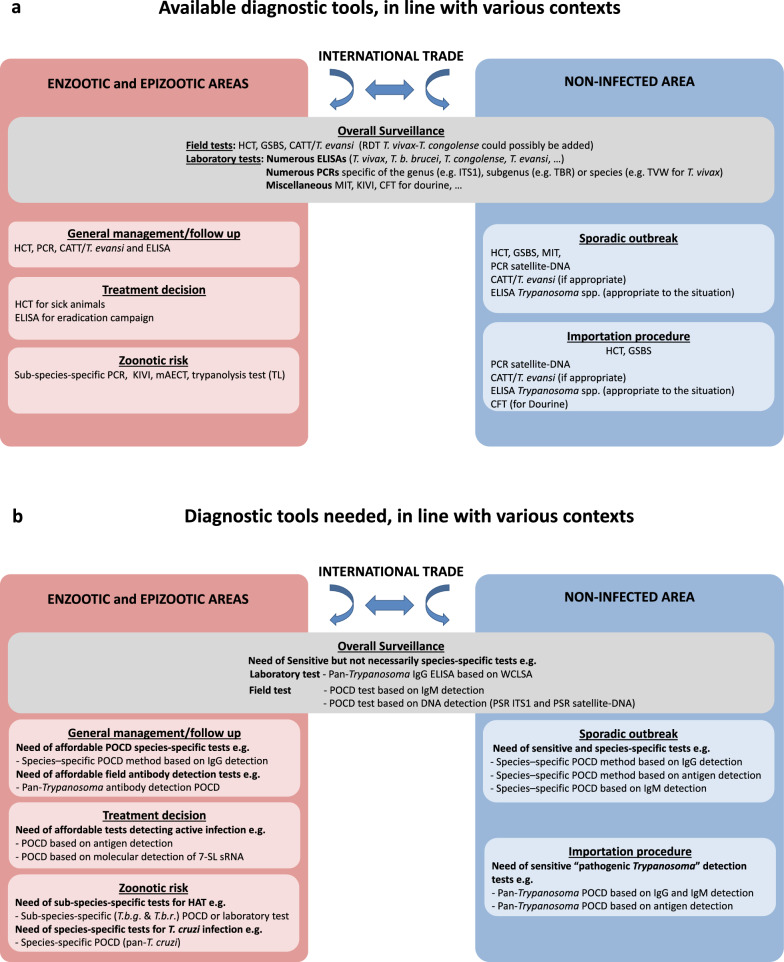
Schematic overview of current diagnostic tools for animal trypanosomoses (**a**) and those needed to improve the diagnosis of animal trypanosomoses (**b**). Diagnostic tests are grouped according to the main epidemiological contexts in which they can be used (enzootic, epizootic areas, non-infected area) and the different situations encountered (general management, treatment decision, zoonotic risk, sporadic outbreak and importation procedures, etc.). New diagnostic test development efforts should benefit from strong interconnections between international research centres, National Agricultural Research Systems and, possibly, private industrial partners, with the aim to facilitate access to biological material from biobanks. Such cooperation will help scientists transform scientific discoveries into field-applicable tests. New tests should first be validated through multi-partner field evaluations before being distributed for large-scale field applications. These developments will need to be supported by updated international recommendations and further national regulations in keeping with epidemiological contexts, to ensure the rapid and widespread adoption of recently-developed tests. See Abbreviation List for the full description of each abbreviation

The diagnostic tools needed can only be defined with regard to the objectives of diagnosis and, thus, most of the time, with regard to the epizootiological situations faced by the end users of the diagnosis results. Therefore, we propose an overview of currently available and advisable methods based on users’ objectives (see Fig. 2a).

### Controlling ATAO in enzootic areas where there is no PCP

When no PCP has been initiated in enzootic areas, nagana control consists in: (i) detecting and treating sick animals; and (ii) applying prevention measures. At an individual level, diagnostic methods should be applied to confirm the clinical suspicion of trypanosomosis and end with a decision on treatment. The clinical evolution of trypanosome infection in an animal may range from subclinical to fatal issues, so a distinction must be made between being a trypanosome carrier and being sick due to trypanosome infection. In enzootic areas, serological evidence of IgG should not lead to a treatment decision, especially in highly enzootic situations where more than 50–70% of the animals are seropositive. Most of these seropositive animals are healthy (asymptomatic carriers or self-cured animals), so no trypanocide treatment is required. However, sick seropositive animals may suffer from other afflictions (such as haemonchosis, anaplasmosis or plant intoxication), which need a differential diagnosis followed by appropriate treatment. Therefore, a decision to treat with a trypanocide requires the establishment of a causal relationship between the trypanosome infection and clinical signs, which is a matter for veterinary medicine.

Asymptomatic carriers may fail to control the infection, and clinical signs due to the trypanosome infection may reappear in cases of immune failure or parasite overload (high seasonal pressure by trypanosome vectors). Therefore, a complementary diagnosis is required in addition to seropositivity to support the clinical signs of trypanosome infection (e.g. anaemia, fever, oedema, etc.). Thus, evidence of recent trypanosome circulation in the blood must be obtained, for example by HCT that can easily detect high parasitaemia, by seeking trypanosome antigens (no test available so far), or by examining DNA (e.g. PCR). In addition, the detection of IgM against trypanosome antigens could assist the diagnosis since this immunoglobulin type reflects recent circulation of the parasite in the blood. Unfortunately, only one CATT exists for animal trypanosomes (for *T. evansi*).

Point-of-care tests (or point-of-care diagnostics) applicable in the field and able to confirm the recent circulation of trypanosomes in the blood are needed for ATAO [98]. Sometimes available via mobile laboratory services, parasitological techniques such as HCT can be applied for parasite detection in the field; if positive, “trypanosome sickness” may be supported by a low packed cell volume (PCV) revealed by the HCT. However, in the case of a negative HCT, a low haematocrit value is inconclusive as it may be due to other conditions. In particular, epizootiological situations (such as in Asia), the CATT for *T. evansi* can be processed in the field. However, for other *Trypanosoma* spp., laboratory equipment is required, thus limiting their appeal. A “rapid antibody test” based on recombinant antigens has been commercialised to detect anti-*T. vivax* and -*T. congolense* antibodies. The initially high sensitivity and specificity reported [99] are being questioned by several unpublished studies. In highly enzootic areas, an “antigen detection test” or an “early-antibody detection test” (IgM) would be a reliable treatment decision tool, as these could confirm that trypanosomes are present or have recently been circulating in the bloodstream of the suspect animals. Indeed, the presence of trypanosomes would indicate the host’s incomplete immune control of the infection.

When it comes to prevention measures, diagnosis may not be critical since such actions are generally applied to all the animals in a group before or after a particular event, such as the seasonal peak of vector activity or a transhumance period.

### Surveys and follow-ups of control campaigns in enzootic areas

In areas where ATAO are enzootic (Africa, Latin America and Asia), epidemiological surveys are generally carried out using both parasitological and serological methods. HCT is the preferred parasitological method for its simplicity, rapidity, and cost. Serological methods such as ELISA are well suited to large-scale, low-cost studies. Whenever possible, PCR methods can be used to increase the sensitivity of active detection and/or provide information on a specific taxon, such as for animal reservoirs of human sleeping sickness in Africa. HCT and ELISA are used in Latin America, along with PCR (including *T. cruzi*) for testing livestock or dogs.

While parasitological methods are widely used, PCR is restricted because of the cost of consumables and the need for equipment and technical skills. These methods are helpful for detecting active infection, but their sensitivity is limited due to fluctuating blood parasitaemia levels. An antibody detection method appears to be better suited. It should be applicable in a large-scale investigation of the prevalence and when a programme ultimately aiming at elimination, such as the PCP, is put in place. In addition, it should allow a follow-up of the prevalence over time. The antibody ELISA for IgG detection meets this need. Depending on the *Trypanosoma* species, a set of one to three ELISAs can be used, including an ELISA for *T. vivax*, one for *T. congolense* savannah and one for *T. evansi* or *T. brucei brucei*, the results being remarkably similar whichever of the two latter antigens is used [17, 100]. When efficient control methods are applied, such serological follow-ups will help to demonstrate a decreasing prevalence.

However, antibody persistence, which has been estimated at around 3–4 months after self-cure or treatment [7], must be taken into consideration to determine the sampling frequency. Reaching the elimination phase of a PCP, animals can be sampled at a regional scale every 6 months to avoid detecting antibodies linked to past infection or treatments. A considerable amount of WCLSAs will be necessary for such large-scale ELISA studies (see "A practical concern: sample preparation and shipment" Section).

To implement control campaigns without the systematic treatment of all animals, field diagnostic tools are helpful for detecting active infection, making treatment decisions and for following up to confirm parasite elimination or to improve the treatment. As seen previously, with the exception of the CATT for *T. evansi*, no antibody detection POCD is currently available, so the methods mentioned above must be applied (HCT/PCR for active infection detection and ELISA for IgG detection), noting the severe drawback of the time needed to obtain ELISA and PCR results. The following POCD tests would be helpful: (i) an antigen detection POCD to identify carriers for treatment decisions; and (ii) an antibody detection POCD to detect asymptomatic carriers or confirm treatment efficacy.

### International movement of animals

The WOAH suggests safe trading conditions for animals imported to a non-enzootic country from an enzootic country (see the Terrestrial Code in "Future prospects" Section). The WOAH Terrestrial Manual already has chapters on nagana and dourine [101], and a chapter on surra is ongoing. However, some hosts and/or pathogens, such as *T. cruzi*, have not been considered in the Terrestrial Code because the chapters are devoted to the most prevalent and/or notifiable animal diseases. Health authorities should therefore remain watchful and decide on health precautions before allowing the importation of potential carriers. The surra outbreaks in Spain and France in the early 2000s should make us aware of the ability of trypanosomes to hide in apparently healthy hosts [1, 102]. The detection of a *T. cruzi* infection in a Texan horse with neurological signs [62] also calls for more awareness of *T. cruzi* infections in livestock, especially horses, and the threat represented by the international movements of animals living in *T. cruzi* areas [103].

The rules for importing animals from an infected into a non-infected country should be based on multiple requirements: (i) animals must originate from a trypanosomosis-free farm in a free area; and (ii) animals must be negative for a series of parasitological, molecular and serological diagnostic tests, as stated in preceding sections and in the WOAH Terrestrial Manual [101]. The most critical and sensitive test would be IgG detection by ELISA, because IgG is a stable immunoglobulin providing sensitive and reliable detection. Furthermore, because the course of positive seroconversion takes between 10 and 20 days, two ELISAs should be performed at a 1-month interval before exportation and during the quarantine period imposed by the importing country. These steps will ensure that apparently healthy animals are neither cryptic carriers nor recently infected within the incubation period. In fact, the stress generated by the transport and introduction of animals into a new environment can trigger the development or relapse of a parasitic infection. This is the reason why a quarantine period is required before animals can be released into a disease-free area. To increase the reliability of diagnosis, parasitological and molecular tests should ideally be carried out in addition to serological tests.

When considering pets such as dogs and cats travelling from enzootic to disease-free areas, the same protocol (2 negative results for parasitological, molecular and serological tests at a 1-month interval) may be proposed. However, the pets could be quarantined at home. Veterinary awareness of the threat of trypanosomosis should be increased through widespread dissemination of this information.

The WOAH-recommended diagnostic method for dourine is the complement fixation test (CFT), which is available in several laboratories, including the WOAH reference laboratory for dourine (Agence Nationale de Sécurité sanitaire de l'alimentation, de l'Environnement et du travail (ANSES), Laboratoire de Santé Animale, Site de Normandie, Unité PhEED, RD675, 14430 Goustranville, France). However, because antigens of the *Trypanozoon* subgenus cross-react, serological tests detecting antibodies to *T. evansi* would probably also detect *T. equiperdum* infection since the antigen used is based on a WCLSA. The CATT also cross-reacts in some *T. equiperdum* infections [104], but it is unknown whether the test would be sensitive enough to detect all *T. equiperdum* infections since a predominant expression of RoTat1.2 variable antigen type (VAT) has not been fully demonstrated in all *T. equiperdum* isolates [105, 106].

### Control of sporadic outbreaks

When a trypanosome is accidentally introduced into a non-enzootic country or area, the priority is to detect the infection as early as possible, with the highest sensitivity. Therefore, similar rules and tools should apply as in the quarantine periods before and just after importation. Three diagnostic tests ought to be used: HCT to detect parasite carriers, molecular tests to increase the sensitivity of HCT and confirm species identification and antibody detection tests to detect asymptomatic carriers. If infected animals are not culled, then antibody tests should be realised after curative treatment, as long as the animals continue to be seropositive and for a minimum of 6 months.

These diagnostic methods were successfully applied during the surra outbreaks in Spain and France [12, 26, 107]. However, in France, all the infected camels became negative to parasitological and molecular tests and seronegative to CATT and ELISA soon after treatment (2–4 months). Despite this, one camel relapsed into infection more than 4 months after a negative seroconversion, suggesting that no diagnostic tests can reliably ascertain the carrier status of the animal [1]. The most likely explanation for such a case is the localisation of the parasite in an extravascular refuge after the first treatments at the origin of a negative seroconversion, followed by reinvasion of the blood some months later (evidenced by HCT, PCR and ELISA testing) [12]. Based on this experience, it is probably safer to cull the animals to eliminate the parasites.

### Investigations into zoonotic trypanosomoses

#### For humans in Africa

Several diagnostic methods are available to detect HAT due to *T. brucei gambiense* or *T. b. rhodesiense* in various samples. such as blood, lymph node juice, cerebrospinal fluid (CSF) and, more recently, dermis exploration [108, 109]. The methodologies used are: (i) direct examination by HCT, GSBS, quantitative buffy coat (QBC) or the miniature anion exchange centrifugation technique (mAECT) [24, 110, 111]; (ii) serological methods, such as the CATT for *T. brucei gambiense* [112] and the trypanolysis test (TL [113, 114]); (iii) the in vitro isolation kit (KIVI) [24, 115]; (iv) molecular detection methods specific to species or subspecies [73, 74, 116]; and even (v) the xenodiagnostic method used in the past [117].

#### For animals in Africa

While the lack of diagnostics for species-specific detection is generally not a problem for controlling animal trypanosomoses (accepted as a “disease complex”), it may become so when an animal sample carries zoonotic parasites. First, one must be aware of the potential for human infection; second, in the generally accepted “One Health” approach, the priority lies in identifying the potential reservoirs of human parasites, such as pigs for *T. b. gambiense* or cattle, pigs and wild mammals for *T. b. rhodesiense* [65, 116, 118–120]. While a *Trypanozoon* infection can be easily characterised at the subgenus level, it is both costly and complex to reach the subspecies level and thus to identify human or non-human parasites [121]. Indeed, PCR based on single-copy genes used to detect *T. b. gambien*se and *T. b. rhodesiense* infections are far less sensitive than satellite DNA amplification. This lack of sensitivity is a severe limitation for species-specific diagnosis [73, 116]. A sensitive and specific diagnosis is thus required but will be challenging to develop.

#### For animals in Latin America

Investigations into wild and domestic reservoirs of *T. cruzi* can be carried out using molecular and serological tools. Since molecular methods are expensive, a serological tool such as an ELISA would be more cost-effective for screening animal populations, but still pricey. The presence of marked cross-reactions with *T. evansi* antigens in an ELISA could motivate a safe and cost-effective option to simultaneously detect antibodies to *T. evansi*, *T. cruzi* and *Leishmania* spp. in both humans and animals [20]. Therefore, an ELISA using *T. evansi* WCLSA could be used for preliminary screenings [20, 54]. This system could be used for the large-scale screening of human blood banks. However, under the pressure of test manufacturers, recombinant rather than native antigens tend to be preferred. In potential reservoirs of *T. cruzi*, this serological screening can be used to define Trypanosomatidae infections. A *T. cruzi* infection can be confirmed by PCR using TCZ primers targeting satellite DNA [29]. Such a confirmation protocol was used to detect a *T. cruzi* infection in a Texan quarter horse [62]. The latter study highlights the risk of *T. cruzi* infection among humans in the USA and the associated risk of the international circulation of *T. cruzi* by travelling horses. Alternatively, species-specific serological tests for *T. cruzi* could be developed based on recombinant antigens if cross-reactions with other Trypanosomatidae are considered (*T. evansi* and *Leishmania* spp.). Care must be taken when handling animal samples containing zoonotic parasites such as *T. cruzi* due to their potential to infect humans. As dogs and horses live in close proximity to humans, the risk of *T. cruzi* being transmitted to humans must be taken seriously, knowing that butchering and consumption of raw horse meat is still a habit in some communities.

#### Atypical human infections by animal trypanosomes

Some animal *Trypanosoma* spp. occasionally infect humans, requiring the development of suitable diagnostic methods. Atypical human infections by animal trypanosomes mainly involve *T. lewisi* and *T. evansi* [122, 123]. Tools to investigate *T. evansi* infections in humans are similar to those applied to animals. However, identification of the species is not always conclusive, especially in Africa, where other subspecies of *Trypanozoon* occur in humans. A PCR method based on the amplification of ITS1 (a moderately repeated sequence of around 500–800 repeats) has been developed to detect *T. lewisi* with medium sensitivity [124]. A more sensitive method is needed as well as serological methods for screening human populations. Attempts have been made to develop an ELISA for *T. lewisi,* but further work leading to validation is required [125]; because of the unknown incidence in humans, this could be a serious challenge.

## A practical concern: sample preparation and shipment

The preliminary steps before diagnostic procedures include sample collection, packaging, shipment, storage and test preparation. Samples must be collected in clean or sterile conditions to avoid bacterial contamination that may affect test results and to prevent human contamination by zoonotic pathogens present in animal samples.

Blood is the most common sample for animal trypanosome or trypanosomosis detection. Crude blood is used for parasitological techniques (HCT/PCV, GSBS, QBC, MIT); DNA purification is necessary for molecular techniques (DNA extraction from blood or buffy coat); and cell separation is required for serological tests on serum or plasma. A recent review [109] listed other samples used, including lymph node juice, CSF and lachrymal, preputial or vaginal secretions; however, it is not possible to obtain sterile conditions for some of these samples. Blood samples must be cooled slowly and kept cool (2–4 °C) to keep parasites alive and slow down microbial proliferation. Samples must be warmed up for HCT, making the parasite more active and visible under a microscope. In all cases, the parasitological diagnosis must be processed as soon as possible, preferably within 2 h of blood collection. Indeed, sensitivity will be affected by delayed examination since parasites will be killed ex vivo by the immune system (phagocytosis, complement lysis, etc.) or be exhausted by a lack of metabolites (e.g. glucose) and will thus not be detected in HCT and MIT [126].

When POCD tests are available (e.g. HCT, CATT), no other step is required. Otherwise, samples must be transported under cold conditions to the primary laboratory. Parasitological tests and buffy coat preparation for PCR should be carried out as soon as possible, preferably within 2–6 h of blood collection.

When the primary laboratory cannot carry out certain tests, samples have to be packaged and sent to a reference laboratory. For example, FTA^®^ cards (Whatman plc, Maidstone, UK) are effective in preserving genetic material in blood samples [127–129] but filter paper (e.g. Whatman® Grade No. 4 Qualitative Filter Papers) is an alternative for blood drops or a buffy coat (blood dots) for PCR, as well as for serum or plasma (serum dots) for serology [126]. Convincing results have been obtained with PCR, ELISA and even CATT when buffy coat, serum or plasma are recovered from filter papers [126]. Once dried, samples can be sent in an envelope, following recommendations detailed in the WOAH guidelines [126]. The advantages of such packaging conditions are significant since dry samples can travel at room temperature, are lightweight and are not considered to be infectious (https://www.woah.org/en/what-we-do/standards/codes-and-manuals/terrestrial-manual-online-access).

In all instances, the shipment must comply with International Air Transport Association rules and requires exportation and/or importation authorisations from the veterinary services of the countries involved. Furthermore, such samples can only be used for diagnosis. A specific agreement in compliance with the Nagoya Protocol for access and benefit-sharing (https://www.cbd.int/abs/) is required for other purposes. On arrival in a reference laboratory, samples should be stored at + 4 °C or frozen at − 20 °C until processed.

Dry sample shipment is a considerable improvement in animal trypanosome diagnostics that deserves to be broadcasted, as it will help expedite reception of test results by the end users.

## Future prospects

Several promising reports on vaccine candidates against animal trypanosomes have been published [130, 131], including flagellar membrane [132] and beta-tubulin [133], but at the time of writing, no vaccines are yet available. Beta-tubulin and flagellar membrane antigens are potential targets for diagnostic developments.

Global control programmes for animal trypanosomoses are based on: (i) the control of vectors; (ii) diagnosis, which is crucial to control; and (iii) treatment. WOAH reference methods for trypanosomosis diagnosis are reliable, but their performances and availability are limited. Parasitological methods can reveal the infections when parasitaemia is high enough, which is mainly in recent or acute infections and relapses; however, they are only subgenus specific. Serological methods can reveal the contact between hosts and parasites with high sensitivity, and they can also detect chronic and subclinical infections, including asymptomatic carriers; however, they are not species specific and may not indicate ongoing infection. Finally, molecular techniques improve the sensitivity of parasitological methods and their specificity can generate robust epidemiological data.

However, some gaps still exist, including: (i) some methods require highly skilled technicians and fully equipped laboratories; (ii) some specific reagents (e.g. reference serum samples) are not standardised and/or not commercially available (e.g. WCLSAs); (iii) POCD methods are not available to address these issues; and (iv) they are costly, especially molecular techniques. Therefore, improved diagnosis of animal trypanosomosis will require less expensive tests due to the lack of financial resources of rural populations in the most enzootic countries. Furthermore, the methods should ideally be 100% sensitive and 100% species specific. New diagnostic methods will have to overcome the challenges of sensitivity, originating from low parasitaemia levels during infection, and of specificity, avoiding the cross-reactions among salivarian trypanosomes [17, 21, 134, 135], and between salivarian and stercorarian trypanosomes such as *T. cruzi* and *T. evansi* [20]. An illustrated overview of the ideal diagnostic tests is presented in Fig. 2b.

### Pan-*Trypanosoma* antibody detection ELISA for screening and follow-up

For large-scale epidemiological studies and follow-up of ATAO control campaigns, notably under a PCP, the IgG detection method through antibody-ELISA for trypanosomes could be better standardised by in vitro production of trypanosomes. Reagent conservation (coating antigens and reference serum samples) and transport of samples are mainly improved by lyophilisation [136].

#### In Africa

Nagana is a complex disease due to marked immunological cross-reactions between the various species of *Trypanosoma* present [17]. Species-specific serological methods are therefore neither expected nor required for nagana control. It would be thus advisable to develop a single IgG detection ELISA for all pathogenic trypanosomes. Used jointly, WCLSAs from the most dominant parasites (*T. vivax*, *T. brucei* and *T. congolense* type savannah) would provide a sensitive pan-pathogenic *Trypanosoma* antibody detection test. Such tests should be distributed among regional laboratories to support large-scale studies and follow-up. These trypanosomes produced in vitro provide a large-scale, standardised source of antigen. A reference laboratory can supply positive and negative reference serum controls, which can be replaced by local samples selected from regional field samples. The *T. evansi* WCLSA alone, as a cross-reacting trypanosome antigen, may be a good option with application to humans and animals for large-scale low-cost studies of African trypanosomoses.

#### In Latin America

It may not be necessary to discriminate between *T. evansi* and *T. vivax* infections straight away. Additionally, *T. cruzi* infections may be detected using *T. evansi* antigens. Thus, a mixed WCLSA-based ELISA (*T. vivax* and *T. evansi* antigens) could be beneficial. Alternatively, based on the strong multilateral cross-reactions, a single ELISA for *T. evansi* may be used for screening humans and animals. However, to identify animals with a risk for human health (i.e. infected by *T. cruzi*) or horse survival (i.e. infected by *T. evansi* or *T. equiperdum*), more specific, complementary tools would be needed to discriminate between *T. evansi*, *T. equiperdum*, *T. vivax* and *T. cruzi*.

### Species-specific IgG POCD methods

Species-specific IgG POCD methods may be developed by producing recombinant antigens; indeed, although recombinant antigens cannot compete in terms of sensitivity with the large antigenic panel presented by native antigens, they have serious advantages for species-specific antibody tests. Such tests are desirable for the community in charge of tsetse eradication campaigns: (i) in Africa, to specifically detect carriers of tsetse-transmitted trypanosomes (*T. congolense* and *T. brucei*); (ii) in Latin America, to support trypanocide treatment (diminazene aceturate for *T. vivax* vs melarsomine hydrochloride for *T. evansi*); or (iii) wherever appropriate, to identify animals carrying the human pathogens *T. cruzi*, *T. b. gambiense* or *T. b. rhodesiense*. The first step involves identifying species-specific antigens, which is a challenge in itself. The second step is to take up the sensitivity challenge. Selecting a specific antigen will give better specificity but considerably reduces the test’s sensitivity because a single recombinant antigen will address a single or a small number of clones of the host’s humoral immune system (B lymphocytes). Therefore, to improve the sensitivity of recombinant antigen-based antibody detection methods, combining several species-specific recombinant antigens in a single test should be considered [137], including early, late and immuno-dominant antigens. Such a strategy might work for *T. cruzi*, *T. vivax* and *T. congolense* but would probably fail for *T. evansi* versus other *Trypanozoon* species or subspecies (including the distinction of zoonotic subspecies of *T. brucei*), due to the very limited antigenic polymorphism expected in *T. brucei *sensu lato.

Antibody-based diagnostic methods may easily fulfil the cost-effectiveness criterion of POCD for trypanosomoses. Preliminary attempts have been made to identify immunogenic antigens of *T. congolense* and *T. b. gambiense* [138, 139]. A rapid antibody test based on IgG detection has recently been developed, based on the single antigens TcoCB (*T. congolense* cathepsin B-like protease) and TvGM6 (GM6 antigen of *T. vivax*) for *T. congolense* and *T. vivax*, respectively [99]. This is the first commercial POCD brought to the market for animal trypanosomes [99], but the drawback of low sensitivity associated with a single antigen persists, while this POCD remains too costly for large epidemiological studies. Some further attempts were made to develop lateral flow POCD based on recombinant antigens, such as TcoCATL_FL_ (full-length *T. congolense* cathepsin L-like protease) [140] for *T. congolense* and TviCATL (*T. vivax* cathepsin L-like protease) [141] and P310 [142] for *T. vivax*. However, despite promising results, the tests did not reach the commercial stage (T. Coetzer, personal communication) Therefore, it is desirable to increase test sensitivity by combining several recombinant antigens from each targeted species, rather than using single molecules. Similar strategies are advisable for other *Trypanosoma* species, such as *T. brucei* and *T. cruzi*.

Finally, attention should be paid to the development of native antigens produced in vitro since they promise: (i) higher sensitivity, thanks to the large panel of antigens considered; (ii) standardisation of antigen production; and (iii) the ethical advantage of in vitro versus in vivo production. Such POCDs would support a treatment decision (in enzootic areas with low prevalence) and/or allow both treatment decisions and post-treatment follow-up in the field in a PCP context.

### IgM POCD methods

Immunoglobulin M (sometimes referred to as “agglutinins”) is a pentameric immunoglobulin, with 10 antigen-binding sites and five complement fixation sites (Fc portions), held together by disulphide bonds and J chains [143]. This structure allows IgM to capture several epitopes, creating agglutinations, which is a network made up of several antigens (protein, particles or cells) and IgM molecules. In addition to this tridimensional structure, IgM can convey increased avidity for binding an antigen [144]. These molecules have multiple specific characteristics since they (i) are produced early at the onset of infection, (ii) have a short half-life and (iii) have powerful agglutinating and neutralising properties [145, 146]. Due to their early production, they are suitable targets for early detection of antibody responses to a pathogen. The trypanosome membrane is covered by variable surface glycoproteins (VSGs); once the host’s immune system recognises a VAT, parasites displaying this VAT are killed and phagocytised by the immune system. Trypanosomes harbouring another VAT then multiply and generate a new peak of parasitaemia, triggering the generation of new IgM, and so forth. This phenomenon is behind the waves of parasitaemia and fever observed in salivarian trypanosomosis.

These cyclical manifestations generate a series of IgM productions, inducing a somehow continuous and renewed presence of IgM in the host’s blood during the parasitaemic phase of the infection. These characteristics make IgM a good target for trypanosomosis diagnosis. However, their neutralising properties (linked to their high affinity for the complement) [145, 146] induce the formation of immune complexes, which are phagocytised and lead to variable levels of free IgMs in the serum of infected animals. Indeed, IgM levels fluctuate daily during an ongoing animal infection, as observed using the CATT for *T. evansi* [8, 75]. Consequently, IgM detection has proven unreliable if processed only once in an animal, a bias easily solved by serial sampling. For animal trypanosomoses, only the CATT for *T. evansi* infections [147] has been developed with an antigen expressed in the early stage of most *T. evansi* infections, the dominant VAT RoTat1.2 [147, 148]. It can be speculated that, in mechanically transmitted species such as *T. vivax* in Latin America, a similar mechanism leading to a predominant VAT might occur, thus prompting development of a CATT for *T. vivax*. In contrast, due to the genetic recombination of trypanosomes that can occur in tsetse flies [149], it is unlikely that the first VAT expressed by a *T. vivax* (or *T. congolense* or *T. brucei*) that is cyclically transmitted by tsetse flies would be a predominant one*.*

However, other parasites with clonal proliferation, such as, for example, *T. equiperdum* type OVI and type BoTat, or *T. evansi* type B [66], probably also have a predominant VAT. Therefore, attempts to develop POCD, such as CATT for *T. vivax* (in Latin America) and CATT for *T. equiperdum*, or even CATT for *T. brucei* subspecies, could be developed, although highly predominant VATs might not be expressed in all *Trypanosoma* spp. Indeed, these VATs probably became predominant in *T. evansi* type A (a mutant of *T. brucei*) because of genetic deletion [67, 150]. Therefore, investigations into the early VATs of *T. vivax* from Latin America, *T. equiperdum* (type OVI and type BoTat) and *T. evansi* type B would be the first step toward such a development. This kind of approach could also be explored to detect IgM against *T. lewisi*.

### Antigen detection POCD methods

Initial attempts to develop antigen detection tests (also called capture or sandwich tests) for ATAO based on monoclonal antibodies failed three decades ago [151]. Still, tests based on antigen detection would be very valuable for treatment decisions and/or species identification.

The circulation of trypanosome antigens in the blood of a host occurs at the time of parasite invasion. These antigens are probably more stable than the parasites themselves because the host immune system may not immediately neutralise all of the parasitic antigens released into the bloodstream. Therefore, the presence of trypanosome antigens could be considered a sign of incomplete control of the infection, supporting a treatment decision. To develop such tests and ensure their sensitivity, an antigen capture system should jointly target a range of constitutive, excretory-secretory, variable and non-variable species-specific antigens. However, characterising such antigens is challenging and would require a library of well-characterised serum samples. Even if hypothetical, exclusive antigen detection tests specific to subspecies, such as *T. brucei gambiense* and *T. brucei rhodesiense,* or even a pan-*T. cruzi* detection test would be valuable for identifying animals carrying these zoonotic pathogens. In *T. brucei*, such a distinction might be possible based on limited polymorphism. Identifying immunodominant and shared circulating antigens of *T. cruzi*, which exhibits a high polymorphism, could also be challenging.

Consequently, in areas where HAT and ATAO co-exist, it would be preferable to consider them as a disease complex that must be tackled as a whole using a set of tools based on diagnosis, treatment and vector control through a “One Health African trypanosomosis” approach.

### DNA- and RNA-based diagnostic methods

Point-of-care diagnostics based on DNA amplification, such as LAMP [152], have been developed, but have not been successfully applied in the field. New developments, such as RPA-LFD or even PSR [37] are promising. Recent studies have demonstrated a high amount of small trypanosome RNA in the serum of cattle with active infections [39, 153]. This 26-nucleotide RNA fragment in the blood or serum is derived from the non-coding 7SL RNA of the peptide signal recognition particle. They are potential disease biomarkers because of their stability, their accessibility and the availability of technologies to detect them [153]. The nucleotide sequence of 7SL RNA-derived small RNA, which is conserved among *Trypanosoma* spp., can allow discrimination between *T. brucei, T. congolense* and *T. vivax.* This methodology could be used before microscopic detection of parasites in the blood during the parasitaemia phase, and during remission periods when no parasites are detectable by microscopy. In addition, the level of small RNA decreases after trypanocide treatments, providing a good prediction of active infection [153] and a robust rationale for developing cheap, sensitive and precise field diagnosis methods for trypanosomoses [153].

In view of the above, the authors suggest the development of: (i) a LAMP POCD technique that could be used in the field, or, better; (ii) recombinase polymerase amplification [154]; or even (iii) a lateral flow dipstick, which may be a promising technology for the future [155] and which could emerge from a new technique (e.g. SHERLOCK [specific high-sensitivity enzymatic reporter unlocking[), based on CRISPR (clustered regularly interspaced short palindromic repeats) technology [156, 157], whose application to HAT is already being investigated (https://research.pasteur.fr/fr/project/sherlock4hat/). However, the success strongly depends on a low cost for end users, whether targeting animals or humans.

Alternatively, through the development of a portable next-generation sequencer (e.g. MinION platform; Oxford Nanopore Technologies Ltd., Oxford, UK) connected to a laptop computer via a USB cable, DNA sequencing can now be performed anywhere, even in the field [158]. This technique, which can detect and type virtually any trypanosome species, remains limited by the cost of sequencing, the expertise required for processing and analysing the sequences and by the fact that relatively high parasitaemia levels are required to obtain exploitable DNA sequences [159]. However, the standardisation of these analysis processes, advances in technology and reductions in cost could in the future lead to more general use of direct sequencing in suspected trypanosomosis cases.

## Conclusion

We have suggested here avenues for research to improve the diagnosis of trypanosomosis in animals and occasionally in humans. Generally speaking, the diagnoses have several applications, advantages and limitations, but face challenges in terms of technology, feasibility and adoption. The characteristics of these developments are summarised in Table 1. Some will be easy to develop since all the parameters, methods and reagents are available, such as a pan-*Trypanosoma* ELISA or POCD based on in vitro*-*produced parasites. Some will require short- or medium-term research plans, such as RNA-based methods or PSR (or even SHERLOCK), for which preliminary supporting work has recently been published [37, 153]. Others may require long-term research before field implementation, such as a card agglutination test for IgM detection targeting *T. vivax*, *T. equiperdum* or *T. lewisi*, provided a dominant early antigen is identified, or a species-specific POCD based on recombinant antigens.

**Table 1:** Overview of promising methods that can be developed to meet the needs of field applicable animal trypanosomosis diagnostic tests

Diagnostic methods	Applications	Advantages	Limitations	Challenges
Pan-*Trypanosoma* IgG ELISA based on WCLSA (mixt *Trypanosoma* spp or *T. evansi* antigens)	­ Large scale epidemiological studies and followups of Trypanosomoses control campaigns	­ Highly sensitive pan-pathogenic *Trypanosoma* antibody detection test	­ Not a species-specific method	­ Produce and lyophilize large scale of WCLSA and reference sera
		­ Potential zoonotic application	­ Requires adaptation of the anti-IgG conjugate to the host species	­ Set up in vitro production of parasites for a well standardized antigen production
	­ Possible application in all geographical areas	­ Potential application to *T. cruzi* in humans and animals	­ May hardly be converted into a POCD	
Species–specific POCD method based on IgG detection for ATAO	­ Support decision-treatment and choice of appropriate treatments	­ Species-specific or even subspecies specific test	­ Speciesor sub-species-specific tests have low sensitivity	­ Identify several antigens being: species-specifics, early and late as well as immuno-dominant
	­ Detect, specifically, carriers of tsetse-transmitted trypanosomes	­ Applicable at field level	­ The use of recombinant antigens could impair the sensitivity of the test	­ Standardize the production of multiple recombinant antigens within a POCD tests
	­ Identify animals carrying human threatening trypanosomes	­ Under a PCP, can support treatment-decision in the field		
POCD based on IgM detection	­ Any situation requiring an ATAO diagnosis	­ The presence of IgMs in the host blood, is continuously renewed during parasitaemic phases	­ Fluctuating levels of IgM along infection (can be resolved by serial sampling of suspected animals)	­ Identify a predominant VAT of clonally reproduced trypanosomes (*T. vivax* mechanically transmitted and *T. equiperdum*…)
POCD based on antigen detection	­ Sensitive detection of active infection	­ Amount of circulating antigens more stable than the parasitaemia itself	­ Low levels of circulating species-specific antigens in the host's blood	­ Identify and produce suitable antigens despite the high costs of preliminary tests
	­ Treatment-decision	­ Presence of antigens in a sufficient amount, can support a treatment-decision	­ Identification of species-specific and exclusive antigens for certain species remains hypothetic	­ For high sensitivity, mixed of constitutive, excretory-secretory, variable and non-variable species-specific antigens is required
	­ Species-specific identification			
POCD based on molecular detection of 7-SL sRNA	­ Large scale epidemiological studies and follow-ups of ATAO control campaigns	­ 7SL sRNA signal detected at high levels in the serum of actively infected animals	­ The sensitivity of this technique remains to be confirmed in field samples for actively, chronically or sub-clinically infected animals	­ Apply this technique in POCD while maintaining sufficient sensitivity and acceptable cost
	­ Detection of active infection	­ Allows distinguishing *T. brucei*, *T. congolense* and *T. vivax*		
	­ Treatment-decision	­ Early clearance of 7SL sRNA levels allow detecting active infection and demonstrating treatment efficacy	­ Cost of the technique may not be low enough for a large adoption	
	­ Species-specific identification			
Visual PSR assay	­ Large scale epidemiological studies and follow-ups of ATAO control campaigns	­ Rapid visual test	­ The sensitivity of this technique remains to be confirmed in field samples for actively, chronically or sub-clinically infected animals	­ Obtain a sufficient sensitivity and an acceptable cost; ­ Being able to develop specific PSRs for al pathogenic *Trypanosoma* species
	­ Detection of active infection	­ Applicable at field level		
	­ Treatment-decision	­ Species-specific test	­ Cost of the technique may not be low enough for a large adoption	
	­ Species-specific identification			

At the end of the day, sophisticated and expensive tests are a waste of time and resources, and also contribute to global warming. The task of scientists is therefore to develop new tests to control trypanosomes and trypanosomoses that are accurate, cheap, easy to use and quick to implement to ensure their widespread use.

## Data Availability

All the data generated or analysed during this study are included in this published article.

## Abbreviations

AAT: Animal African trypanosomosis
ATAO: Animal trypanosomoses of African origin
CATT: Card agglutination test for trypanosomiasis
CFT: Complement fixation test
CRISPR: Clustered regularly interspaced short palindromic repeats
CSF: Cerebrospinal fluid
ELISA: Enzyme-linked immunosorbent assay
FAO: Food and Agriculture Organisation (United Nations)
GSBS: Giemsa-stained thin blood smear
HAT: Human African trypanosomiasis
HCT: Haematocrit centrifugation technique
IgG: Immunoglobulin G
IgM: Immunoglobulin M
ITS1: Internal transcribed spacer 1
KIVI: Kit for in vitro isolation
LAMP: Loop-mediated isothermal amplification
mAECT: Miniature anion exchange centrifugation technique
MIT: Mouse inoculation test
mRNA: Messenger RNA
NGS: Next-generation sequencing
OD: Optical density
OIE = WOAH: World Organisation for Animal Health
PATTEC: Pan African Tsetse and Trypanosomosis Eradication Campaign
PCP: Progressive control pathway
PCV: Packed cell volume
POCD: Point-of-care diagnostics
PSR: Polymerase spiral reaction
QBC: Quantitative buffy coat
qPCR: Quantitative PCR
RoTat: Rode *Trypanozoon* antigen type
RPA-LFD: Recombinant polymerase amplification with lateral flow dipstick
SHERLOCK: Specific high-sensitivity enzymatic reporter unlocking
SL-RNA: Spliced leader trypanosome RNA
TL: Trypanolysis test
UV: Ultraviolet
VAT: Variable antigen type
VSG: Variable surface glycoprotein
WCLSA: Whole cell lysate soluble antigen
7SL: A signal recognition particle
